# The Use of RelocaTE and Unassembled Short Reads to Produce High-Resolution Snapshots of Transposable Element Generated Diversity in Rice

**DOI:** 10.1534/g3.112.005348

**Published:** 2013-06-01

**Authors:** Sofia M. C. Robb, Lu Lu, Elizabeth Valencia, James M. Burnette, Yutaka Okumoto, Susan R. Wessler, Jason E. Stajich

**Affiliations:** * Department of Plant Pathology & Microbiology, University of California-Riverside, California 92521; † Institute for Integrative Genome Biology, University of California-Riverside, California 92521; ‡ Department of Botany & Plant Sciences, University of California-Riverside, California 92521; § College of Natural and Agricultural Sciences, University of California-Riverside, California 92521; ** Graduate School of Agriculture, Kyoto University, Kitashirakawa-oiwake Sakyo, Kyoto, Kyoto 606-8502, Japan

**Keywords:** transposable element, rice, genomics, bioinformatics, NGS

## Abstract

Transposable elements (TEs) are dynamic components of genomes that often vary in copy number among members of the same species. With the advent of next-generation sequencing TE insertion-site polymorphism can be examined at an unprecedented level of detail when combined with easy-to-use bioinformatics software. Here we report a new tool, RelocaTE, that rapidly identifies specific TE insertions that are either polymorphic or shared between a reference and unassembled next-generation sequencing reads. Furthermore, a novel companion tool, CharacTErizer, exploits the depth of coverage to classify genotypes of nonreference insertions as homozygous, heterozygous or, when analyzing an active TE family, as rare somatic insertion or excision events. It does this by comparing the numbers of RelocaTE aligned reads to reads that map to the same genomic position without the TE. Although RelocaTE and CharacTErizer can be used for any TE, they were developed to analyze the very active *mPing* element which is undergoing massive amplification in specific strains of *Oryza sativa* (rice). Three individuals of one of these strains, A123, were resequenced and analyzed for *mPing* insertion site polymorphisms. The majority of *mPing* insertions found (~97%) are not present in the reference, and two siblings from a self-crossed of this strain were found to share only ~90% of their insertions. Private insertions are primarily heterozygous but include both homozygous and predicted somatic insertions. The reliability of the predicted genotypes was validated by polymerase chain reaction.

Transposable elements (TEs) are fragments of DNA that often increase their copy number as they move from one genomic location to another. With the advent of whole-genome sequencing, it has become clear that TEs comprise the largest fraction of the genomes of plants and animals. For example, TEs comprise more than half of mammalian genomes and as much as 85% of some plant genomes (Lander *et al.* 2001; Waterston *et al.* 2002; Schnable *et al.* 2009).

To understand how TEs increase in copy number without killing their host, we are characterizing the amplification of an extremely active element called *mPing* in rice (*Oryza sativa*) (Jiang *et al.* 2003). Although there are 51 copies of the 430-bp *mPing* element in the reference Nipponbare (NB) genome (Naito *et al.* 2006), several rice strains were identified with hundreds of copies. Furthermore, *mPing* is still actively transposing in these high copy strains and accumulating dozens of insertions per plant per generation (Naito *et al.* 2006). In a previous study we used vectorette polymerase chain reaction (PCR) coupled with 454 sequencing to characterize almost 1700 insertion sites of *mPing* in a small population of strain HEG4 and determined that the element had a preference for insertion into promoter regions (Naito *et al.* 2009).

Given the rapid amplification of this element, we sought to detect *mPing* insertion-site polymorphism in very closely related individuals. Rice is well suited for this type of analysis as it is propagated by self-pollination and most lines are highly inbred. We identified *mPing* insertion sites in multiple progeny by comparing unassembled next-generation sequencing (NGS) reads to the reference NB genome. In addition, we used the depth of coverage to determine the genotype of each site characterizing the alleles as homozygous (old), heterozygous (new), or somatic insertions in each individual.

The sheer abundance of TEs has made accurate genome assembly (Pop 2009) and annotation (Yandell and Ence 2012), challenging, especially with short read technologies.. To identify TE insertion sites in assembled sequences, most studies (Fernández-Medina *et al.* 2011; Lu *et al.* 2011; Labbe *et al.* 2012) use tools such as RepeatMasker, BLAST, and BLAT (Smit *et al.* 1996-2010; Altschul *et al.* 1997; Kent 2002). Using the same methodologies to identify genome wide TE insertions in unassembled NGS is difficult due to the small size (~35−100 bp) and the large number of reads (millions). A few tools have been created that use unassembled NGS reads to locate TE insertions (Iskow *et al.* 2010; Witherspoon *et al.* 2010; Linheiro and Bergman 2012; Tian *et al.* 2012), but they do not have the capability to characterize the genotype of insertion sites.

To facilitate this comparative analysis of *mPing* insertion sites, we report the development of RelocaTE and a companion tool, CharacTErizer. RelocaTE identifies three different groups of insertion sites for specific TEs: (1) full-length insertions that are present in the reference genome (reference); (2) polymorphic insertions that are present in the unassembled NGS reads and not in the reference (non-reference); and (3) those that are present in both the reference sequence and in the NGS reads (shared). CharacTErizer predicts the genotype of nonreference insertions. We show that these tools are of general use in the identification of any TE insertion site in unassembled NGS reads where a reference genome, TE sequence and the target site duplication (TSD) information is available.

## Materials and Methods

### Library preparation and sequencing

Genomic DNA was extracted using the CTAB method (Huang *et al.* 2000). DNA quantity was checked with a NanoDrop spectrophotometer (NanoDrop products, Wilmington, DE). Sequencing libraries for plants A123-1 and A123-2 were constructed using the Illumina TruSeq DNA Kit version C (Illumina Inc., San Diego, CA) and quantified by Bioanalyzer 2100 (Agilent Technologies, Santa Clara, CA). An alternative thermocycling protocol was used for A123-1 (extended the initial denaturation from 30 sec to 3 min and each subsequent denaturation step from 10 sec to 80 sec) (Aird *et al.* 2011). The library from plant A123-0 was constructed using Illumina Paired-End Sample Sequencing Preparation Kit. Libraries were multiplexed to 10 nM and were sequenced on a HiSeq2000 pair-end 100-bp run in the UCR IIGB Genomics Core Facility (http://illumina.ucr.edu). The Illumina CASAVA 1.8.2 pipeline was used for base calling and de-multiplexing.

### Data processing

All data processing was performed on the HPC Biocluster in the UCR IIGB Bioinformatics core (http://www.bioinformatics.ucr.edu/). Perl processing scripts are available through the RelocaTE package (https://github.com/srobb1/RelocaTE) and as Supporting Information, File S2.

### Identification of nonreference *mPing* insertions using RelocaTE

To identify the location of a nonreference TE insertion, sequence reads containing the TE sequence along with flanking reference sequence must be identified, trimmed of TE sequence and aligned to the reference genome. relocaTE.pl launches a series of scripts which runs, parses, and filters output from BLAT (Kent 2002), Bowtie (Langmead *et al.* 2009), and SAMtools (Li *et al.* 2009) to identify a TE insertion from unassembled short reads.

RelocaTE begins by aligning all short reads to the sequence of a TE with BLAT. In the rice analysis this was the *mPing* sequence (accession number: AB087615). The TE sequence must be supplied in FASTA format along with the TSD sequence in the description region (*e.g.*, for *mPing* TSD = TAA). The sequence of the TSD and its reverse complement are used when mapping the location of an insertion site. If the TSD is not a precise sequence, a regular expression representing a pattern or the length of the TSD can be provided (Table S4). For TSDs in which neither the sequence nor the length is known programs such as LTRharvest (Ellinghaus *et al.* 2008) or ngs_te_mapper (Linheiro and Bergman 2012) can be run to discover the TSD sequence and length. Also multiple runs of RelocaTE with different length TSDs can be used to identify the best sequence pattern or length for a tested TE.

**Figure 1:**
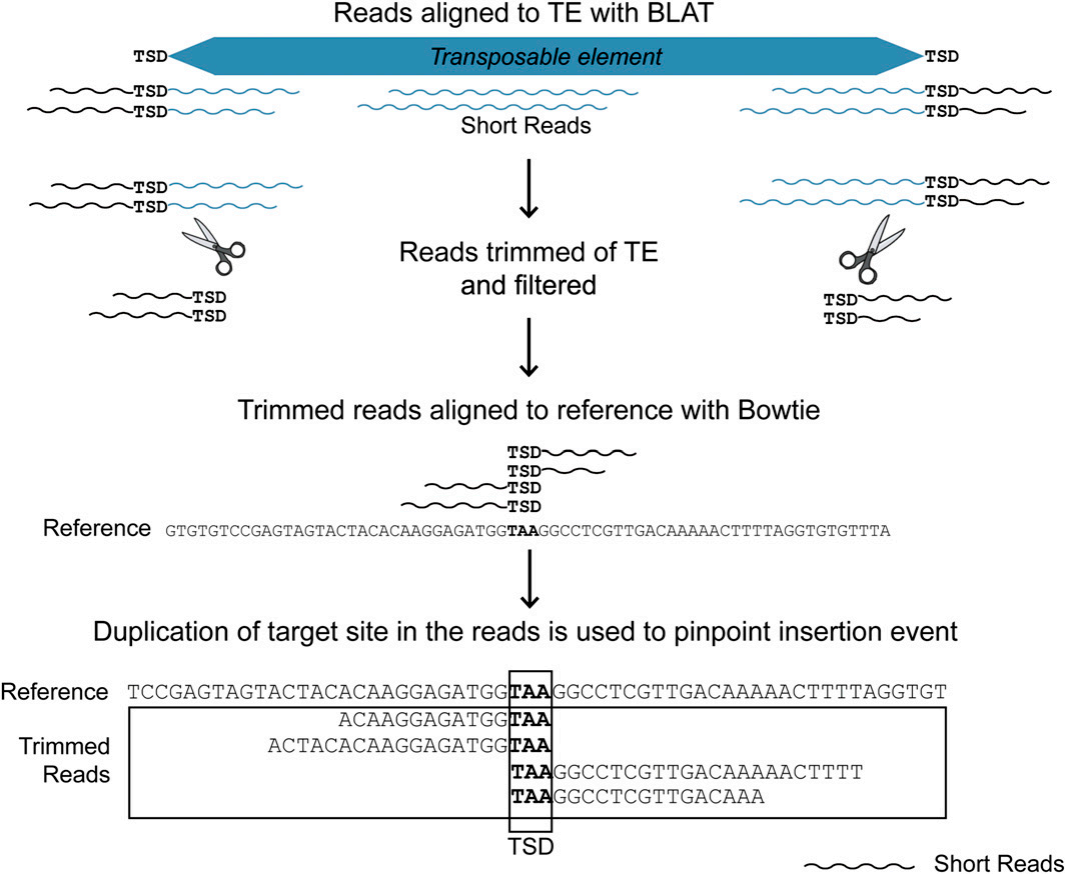
Identification of TE insertion sites by RelocaTE. Reads containing the provided transposable element are retrieved by BLAT, trimmed to remove TE sequence, and aligned to the reference genome with Bowtie to identify the insertion site in the rice genome. The aligned reads must overlap by the exact sequence of the target site duplication (TSD, TAA for *mPing*) that is generated during TE insertion. The location of the insertion site is reported as the coordinate of the last base pair of the target site in the reference sequence.

By default, RelocaTE runs BLAT with a tile size of 7 and a minimum score of 10 to allow for alignments of short reads that produce an alignment with a minimal length of 10. These parameters can be adjusted in the command line options of RelocaTE. Reads that do not overlap with either the first or last nucleotide of the TE or have more mismatches than allowed by the mismatch cutoff are filtered. Total mismatches are divided by the length of the nucleotides that aligned to the TE (matches plus mismatches), and this total must be less than or equal to the user-supplied mismatch allowance (default = 0 for perfect alignments). Matching reads are trimmed to remove any portion that aligned to the TE, and a note of which end of the read was trimmed of TE sequence is appended to the sequence name (start or end). Reads that only match the TE are filtered, as they do not provide any flanking genomic sequence information. Only the trimmed reads which are longer than the minimum allowed length (default = 10) are retained and then aligned to the reference genome with Bowtie using the –best option (Figure 1) to identify flanking regions of the TE insertion.

**Figure 2:**
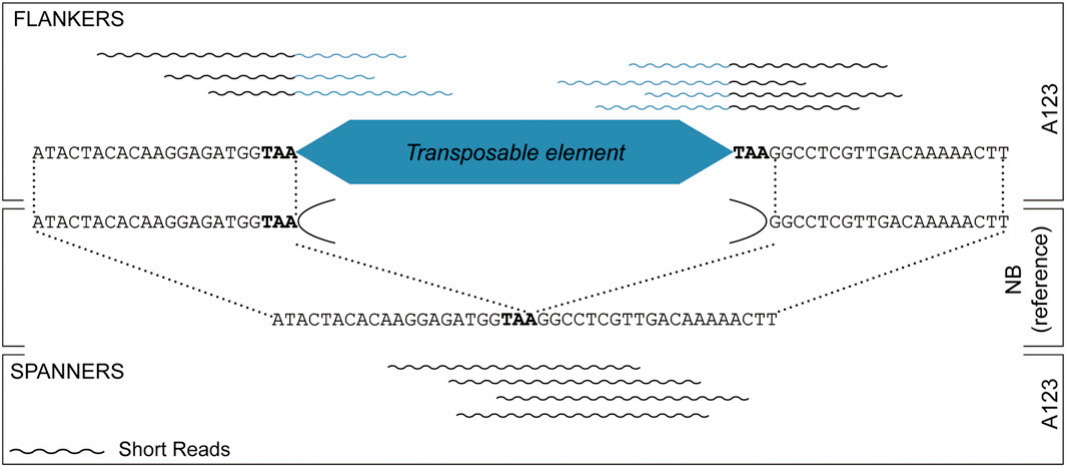
RelocaTE uses flanker and spanner short reads to identify transposable element insertions in the reference NB genome. FLANKERS are reads that overlap the 5′ or 3′ ends of the TE. SPANNERS are reads that align to the same location in the reference genome but containing no TE sequence.

Reads that align to the reference genome (for *Oryza sativa*: MSU release 7) after trimming for TE matching sequence are termed flankers (Figure 2). If paired reads are available, the reads will be aligned individually and again as pairs. Paired reads are not required but may help to identify insertions in repetitive regions. All but one of duplicate alignments will be filtered to avoid redundancy caused by aligning once as individual reads and again as paired. Alignments with more than three mismatches or that do not uniquely map to the reference genome are filtered. Only reads that align to the genome with the appropriate orientation with respect to the trimmed TE sequence are retained. Bowtie alignments are sorted by reference sequence name and then by coordinates to enable fast lookup of the reads. Reads are clustered based on an overlapping alignment to the reference and all clustered reads are labeled as putatively belonging to a single insertion event. Each read is inspected for the presence of the specified TSD.

Finally, for RelocaTE to call a nonreference insertion event, there must be two or more reads, at least one left flanker and one right flanker, with a perfect overlap of the TSD, in the correct orientation, when the reads are aligned to the the reference. The coordinate of the insertion is noted as the coordinates of the target site (Bergman 2012). Because reads that align to more than one location in the genome are discarded, it is currently not possible to identify TE insertions that occur within other repetitive elements that exist in the reference genome.

### Classification of nonreference insertion site genotypes with CharacTErizer

CharacTErizer is used to classify nonreference insertions as heterozygous, homozygous, or somatic using flankers and spanners (Figure 2). characTErizer.pl requires two files to classify the insertion sites, the first listing the locations of all nonreference insertions (determined by flankers) and the second containing alignments of potential spanners to the genome. The first is a tab-delimited output file from RelocaTE and the second is a BAM file of the reads, not trimmed of the TE sequence, aligned to the reference genome (or reference chromosome). The BAM file used here was generated by process_raw_reads.pl.

The use of process_raw_reads.pl is optional and any BWA generated BAM file can be supplied to CharacTErizer. process_raw_reads.pl is used with the input FASTQ files from the sequencer to automate the quality trimming and alignment to the reference. To parallelize data processing, the FASTQ files are split into smaller files of one million reads per file with this same script. Each smaller partition file can be processed in parallel on a multi node HPC cluster. The process_raw_reads.pl filters the sequences with the FASTX-Toolkit (http://hannonlab.cshl.edu/fastx_toolkit/) (fastq_quality_trimmer -l 50 -t 20 and fastq_quality_filter -q 20 -p 80), checks for properly mated sequence pairs followed by alignment to the reference genome with BWA (Li and Durbin 2009) (bwa aln, bwa sampe). Finally, the BWA SAM output is converted to BAM files. Alternatively, the -q option in BWA can be used in place of FASTX-Toolkit filtering.

**Figure 3:**
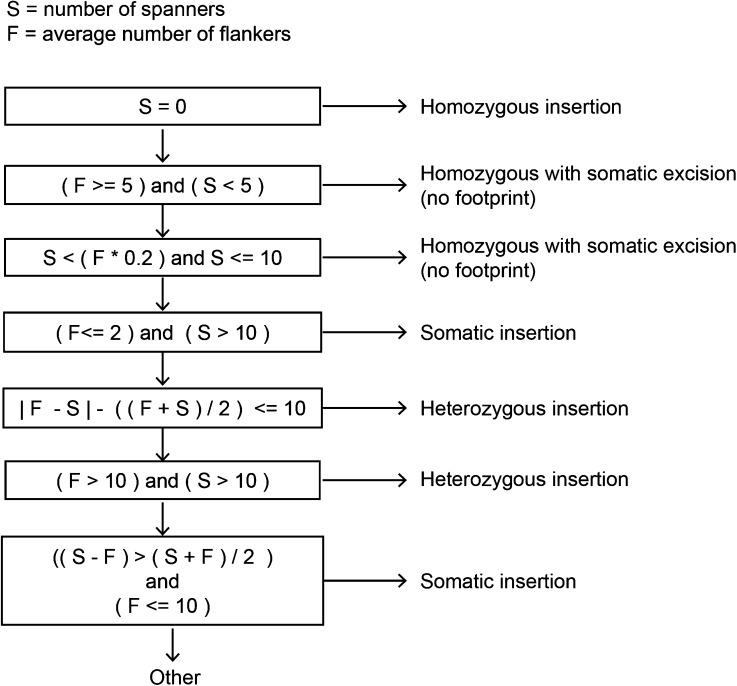
Classification of insertion sites. Flowchart of the conditions required for identifying an insertion as homozygous, homozygous with a somatic excision event, heterozygous, or a somatic insertion. Insertions that do not meet any of the criteria are called “other.”

CharacTErizer compares the number of flankers (reads which flank an insertion site and contain partial TE sequence) to spanners (reads which cover an insertion site and contain no TE sequence). The average number of flankers is used because one insertion site is defined by the presence of flanking reads at both ends. The average number of flankers will be referred to as only ‘flankers’ and the sum of spanners will be referred to as only “spanners.” For each insertion site, the ratio of flankers to spanners is analyzed in a progressive order (Figure 3) to find the first met condition.

i) A homozygous insertion is defined as sites with all flankers and no spanners.
ii) A homozygous site with a somatic excision event (no footprint) is one with five or more flankers and less than five spanners
iii) A homozygous site with a somatic excision event (no footprint) is also defined as a site with 20% fewer spanners than flankers but no more than 10 spanners.
iv) A somatic insertion is a site with no more than 2 flankers and more than 10 spanners.
v) A heterozygous insertion is a site in which the relative difference between flankers and spanners is at most 10 more than the average number of reads used to classify the insertion.
vi) A heterozygous insertion is also a site in which both spanners and flankers total more than 10.
vii) A somatic insertion is also a site in which there are 10 or fewer flankers and the relative difference of spanners and flankers is greater than the average number of reads used to classify the insertion.
viii) A site with any unspecified ratio of flankers and spanners is classified as “other.”

To predict somatic excision events that leave a footprint, CharacTEizer processes alignments of reads to the reference by running SAMtools mpileup to identify nucleotide insertions and deletions. A footprint is identified if the insertion or deletion is located near the TE insertion location (± TSD length).

### Identification of reference, reference-only, and shared insertion locations

RelocaTE will query and report the location of a TE in the reference genome, those insertions also present in the NGS reads, and insertions private to the reference (reference-only) when running relocaTE.pl with the –r 1 command-line option. The positions of full-length TEs in the reference are found by searching the provided TE sequence against the reference genome with the sequence search tool BLAT. Only alignments very similar in length to the query TE (±10%), with less than 10% mismatches over the entire length of the TE are considered to be insertions. These TE site locations in the reference genome are combined with the alignment of TE-trimmed reads to classify sites as shared or reference-only (absence of aligned reads). Reference-only sites are inferred by a lack of reads overlapping ends of the full-length TE in the resequenced strain that could reflect excision in the compared strains, a reference-specific insertion, or lack of sequence coverage. Results are provided in a GFF-formatted report.

### Benchmarking speed

A single library of 120,865,611 reads (32× fold coverage) was divided into 121 files and analyzed by RelocaTE. The entire analysis took approximately 20 min when run in parallel using 64 processors. The same analysis analyzed on a single processer had a total run time of 1 hr and 5 min and used 16 Gb of RAM. Output from the analyses was identical and a total of 364 insertions were found.

### Sequence coverage sensitivity

Analysis of the sensitivity of RelocaTE to variations in sequence coverage showed that although rare somatic insertion sites are often missed in lower coverage, the recovery of homozygous sites was robust to 50% reduction in the data. We simulated lower sequencing coverage by reducing the number of A123-0 reads (32×-fold coverage) by 50% and reanalyzing the nonreference *mPing* sites discovered. The total number of insertions dropped from 364 to 324. Of the 41 insertions that were not detected at the lower coverage, 34 (of the original 56) were somatic, five were homozygous (of 238), and two were heterozygous (of 70). The majority of insertions lost were somatic due to their rarity. Thus, the rare somatic insertions are preferentially lost as the depth of coverage decreases.

### PCR verification

Primer3 (Rozen and Skaletsky 2000) was used to design primers that span the predicted insertion site. MyTaq Red Mix (Bioline USA, Inc, Taunton, MA), which contains Taq, buffer, dNTPs, and loading dye, was used for each reaction. Primer sequences and annealing temperatures are provided in Table S3. PCR amplifications were performed with 5−20 ng of genomic DNA in 10- or 15-μL reactions. Cycling parameters were: 1 cycle at 94° for 3 min, 36 cycles at 94° for 15 sec, 55° (majority of reactions) for 15 sec, 72° for 10 sec, and 1 cycle at 72° for 5 min. The reaction was resolved on 1.5% agarose gels with O’GeneRuler 1 kb Plus DNA Ladder (Fermentas).

## Results

### Identification of *mPing* insertions and their locations using RelocaTE

Strain A123, like other strains of rice (*Oryza sativa*), is largely self-pollinating and, as such, highly inbred. However, unlike most other rice strains, A123 harbors hundreds of *mPing* elements that are actively transposing in both somatic and germ cells (Naito *et al.* 2006). Transposition of *mPing* generates an unprecedented level of genome diversity within a single individual and among progeny from a self-cross (Naito *et al.* 2009). The goal of this study is to use the high depth of coverage afforded by NGS to produce high-resolution snapshots of *mPing*-generated diversity in individual plants.

As a first step in the characterization of *mPing* “bursts,” DNA was isolated from three closely related individuals of strain A123 (see *Material and Methods*) and used to generate 100-bp paired-end Illumina reads to depths of coverage of 19- to 72-fold (Table 1). Next, two programs, RelocaTE and a companion tool, CharacTErizer, were developed to predict and classify *mPing* insertion sites in the unassembled reads. To accomplish this, RelocaTE identifies reads that partially align to the ends of a TE but still contain some unique non-TE sequence. The unique sequence flanking the TE is aligned to the reference genome to identify the location of the TE insertion (called flankers) (Figures 1 and 2). The 430nt sequence of *mPing* along with its conserved TSD, TAA, was used to precisely identify polymorphic, nonreference insertion sites. An average of 308 nonreference insertion sites was identified in each of the three individuals (Table 1).

**Table 1:** Sequence coverage and nonreference *mPing* insertions identified by RelocaTE

	A123-0 (32×)*^a^*	A123-1 (19×)*^a^*	A123-2 (72×)*^a^*
Total *mPing* insertions	364	264	295
Homozygous	238	247	247
Heterozygous	70	16	45
Somatic insertion	56	1	3
Nonreference insertions can be further classified as having an excision event			
Somatic excision	25	11	15

a Fold coverage in parentheses is based on the number of filtered reads that map to the reference genome.

### Classification of nonreference *mPing* insertion-site genotypes using CharacTErizer

**Figure 4:**
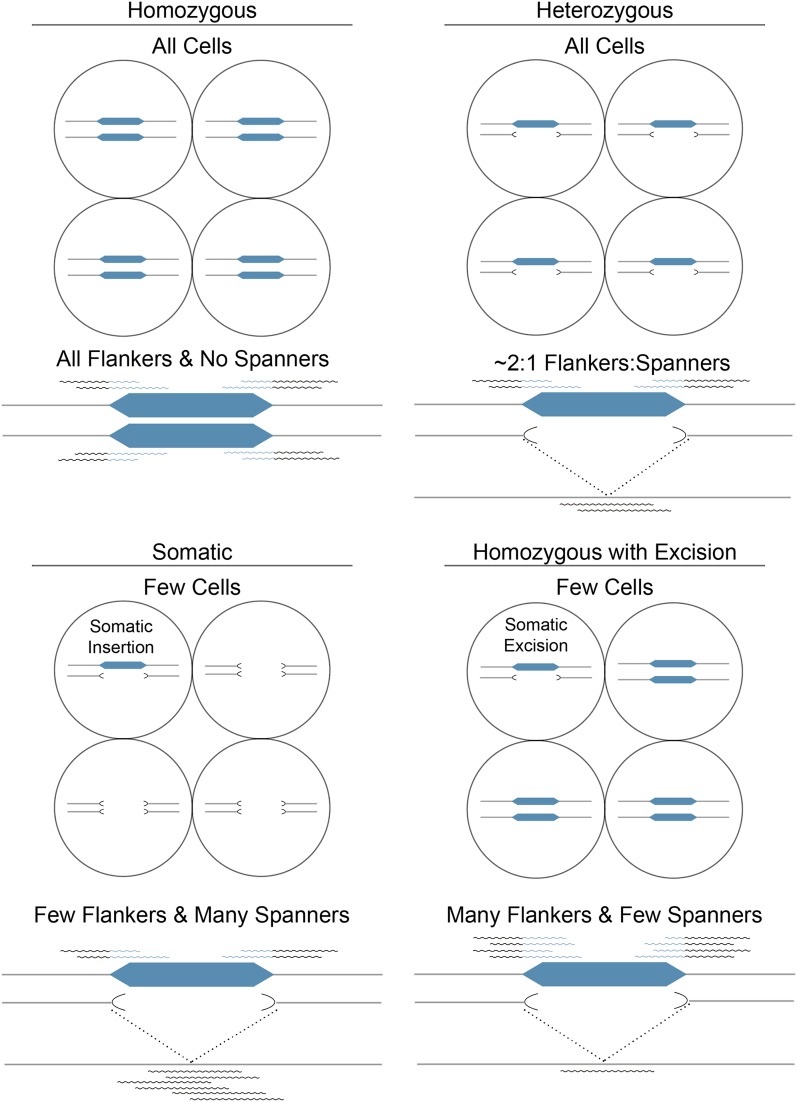
CharacTErizer classification of insertion sites. Alleles are classified based on the ratio of flankers and spanners. Gray lines represent homologous chromosomes in a diploid cell. Homozygous: An insertion that is present in both alleles in all cells. Heterozygous: The ratio of flankers to spanners is approximately 2:1. Somatic: Rare insertions, found in one allele of only a few cells. Homozygous insertion with somatic excision: Somatic excision events can only be identified from homozygous loci.

CharacTErizer is able to detect the presence of two different alleles (with or without the insertion) when used to analyze sequence from a diploid organism like rice. Genotype classification (heterozygous or homozygous) is computed by comparing output from RelocaTE with the alignment of all the reads (before TE trimming) to the reference genome. CharacTErizer compares the number of reads flanking an insertion (flankers) with the number of reads that span the same location in the reference sequence with no gaps (spanners) (Figure 2). The comparison of the number of flankers and spanners can be used to classify the insertion as either homozygous or heterozygous (Figure 4). For example (see Table 2), an *mPing* insertion with many flankers and no or very few spanners would be classified as homozygous because there is evidence for only one allele. In contrast, heterozygous insertion sites are defined by the identification of reads from two different alleles (with or without the insertion), and genome sequencing would sample approximately a 2:1 ratio of flankers and spanners. Using these definitions, CharacTErizer predicted that the vast majority of *mPing* insertions in the three A123 individuals are homozygous.

**Table 2:** CharacTErizer classifications of select *mPing* nonreference insertions in A123-0

CharacTErizer Classification	RelocaTE Insertion Position	Average Flankers	Spanners
Homozygous	Chr8:28359811.0.28359813	27.5	0
	Chr8:763041.0.763043	16.5	0
Heterozygous	Chr7:1924670.0.1924672	13.5	8
	Chr8:21582222.0.21582224	9	9
Somatic insertion	Chr8:23612060.0.23612062	1.5	21
	Chr9:17908908.0.17908910	2	15
Homozygous with a somatic excision event*^a^*	Chr1:1193505.0.1193507	26.5	1
	Chr1:6432234.0.6432236	14.5	2

a No footprint, perfect excision.

High depth of coverage also permits identification by CharacTErizer of the most recent and rare transpositions of *mPing* such as somatic insertions and excisions (Figure 4). Somatic insertions, which exist in only a few cells or in a subset of tissue manifest as a few flankers compared to a much larger number of spanners. A few of these putative events were detected (Table 1). Classification of excision events by CharacTErizer is more complicated. The MITE *mPing* typically excises precisely, leaving no footprint at the excision site (Yang *et al.* 2007). Footprints, which are common among plant (DNA) elements, are identified by a few nucleotides that remain at the insertion site after element excision (Wessler 1988). Precise excision events (where the excision site is restored to the sequence of the reference genome) can be distinguished from homozygous *mPing* loci as many flankers and very few spanners (Table 1, Table S1, and Table S2). CharacTErizer cannot automatically identify precise excision events in loci designated as heterozygous because a perfect excision spanner is indistinguishable from a spanner of an absent TE insertion. In contrast, CharacTErizer can predict the less-frequent imperfect somatic excision events for *mPing* loci classified as homozygous, heterozygous, or somatic because the reference sequence is not restored at the excision site. For all A123 individuals analyzed, excision of *mPing* was predicted to occur at 5–7% of all nonreference insertion sites (Table 1).

### Validating CharacTErizer predictions

We used two strategies to validate the insertion site and genotype predictions of RelocaTE and CharacTErizer. First, we compared the shared and unshared insertion sites in two siblings. Predicted homozygous insertion sites are more likely to be shared than heterozygous sites and predicted somatic events should not be shared. Second, we experimentally validated predicted homozygous and heterozygous insertion sites with PCR using the same tissue from the sequenced individuals.

#### Comparison of *mPing* insertions in two A123 individuals:

**Figure 5:**
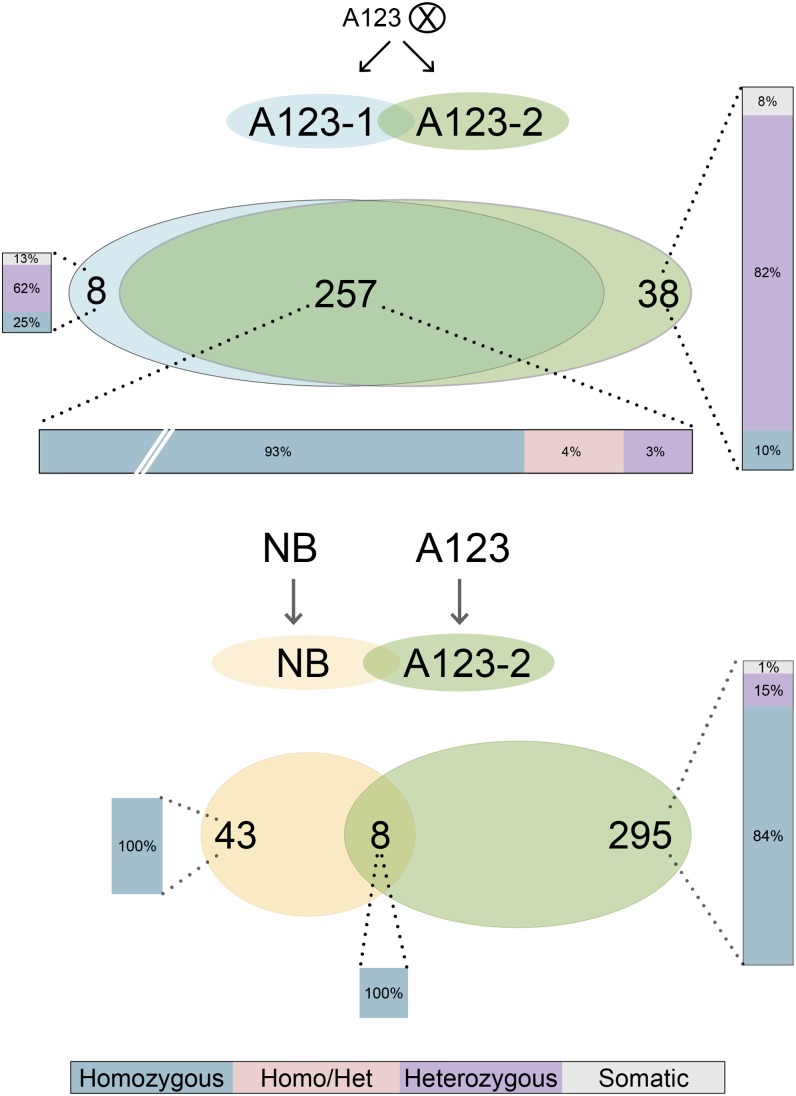
Comparison of *mPing* insertion sites and their genotypes in two A123 siblings. See text for details.

To simplify this analysis, two of the three sequenced individuals, A123-1 and -2, siblings from the same self-fertilized parent, were used for this comparison. As shown in Figure 5, the vast majority of shared insertions are predicted homozygous loci (93%) with the remainder heterozygous in both plants (4%), or heterozygous in one plant and homozygous in the other (3%). Compared with the shared insertions, a much smaller percentage of the unshared insertions are predicted homozygous loci (A123-1, 25%; A123-2, 10%), whereas a much larger percentage are predicted heterozygous (A123-1 62%; A123-2, 82%). Finally, all predicted somatic insertions are unshared (A123-1, 13%; A123-2, 8%). These predicted genotypes are consistent with that of an inbred organism with an actively transposing element in both somatic and germ cells. In contrast, *mPing* is not active in the reference NB genome and all insertion sites are homozygous, including the 8 shared with A123-2. All of the heterozygous insertion sites in A123-2 are unshared, which is also as expected for newer insertions in this active strain (Figure 5).

#### PCR verification of insertions and classifications:

**Figure 6:**
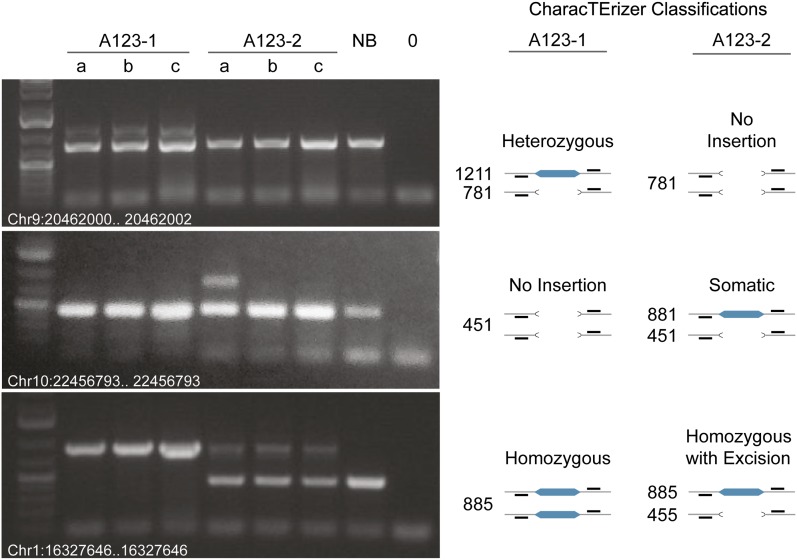
Validation of a subset of predicted *mPing* insertion sites. The presence of each insertion was tested using primers designed for the locus shown and DNA isolated from three different leaves (a, b, and c) of two A123 siblings (A123-1 and A123-2), or from one leaf of NB (no insertion control) or no DNA (negative control) (0). Molecular weight standard is a 1-kb ladder with 500 bp and 1.5 kb bands prominent. The CharacTErizer prediction for each locus is shown on the right with the expected band sizes for the primers used in the PCR validation.

A subset of RelocaTE predicted *mPing* insertion sites were validated by PCR (Table S3). Twenty-six of 26 randomly selected homozygous insertions were confirmed, as indicated by a single amplicon band (data not shown). Twelve of 14 predicted heterozygous sites were validated by the presence of two PCR amplicons bands (see Figure 6 for Chr9:20462000..20462002, other data not shown). Finally, one of the four predicted (rare) somatic insertions (at Chr10:22456791..22456793 in A123-2) was confirmed and appeared as a heterozygous insertion with two amplicon bands in only one of the three leaves of A123-2 (Figure 6). One of the tested insertions predicted at Chr1:16327644..16327646 was classified as homozygous in A123-1 and a homozygous insertion with a somatic excision event in A123-2. This difference in classification was validated by the visualization of only one amplicon (one allele) in A123-1 and two alleles in A123-2 (Figure 6).

### General use of RelocaTE to find TE insertion-site polymorphisms

RelocaTE can be used to identify polymorphic insertion sites for elements other than *mPing*. Individual sequences of three class 2 transposons, *nDart1-1* and *Gaijin* and six retrotransposons, *Dasheng*, *RIRE2_LTR*, *RIRE3A_LTR*, *SPMLIKE*, *COPIA2-LTR*, and *TRUNCATOR* were analyzed for nonreference insertions in A123-2 (Ouyang and Buell 2004; Jurka *et al.* 2005) (Table S4). Variable sequences of defined length (as a Perl regular expression) were used in place of an exact TSD sequence. In order to match TEs that have diverged, different mismatch allowances (0%, 2%, 10%) for identifying TE containing sequence reads were tested. All TEs but *TRUNCATOR* were found to have polymorphic insertions. As the mismatch allowance increases, the number of TE insertion sites identified also increases. RelocaTE output includes a listing of all the non-reference insertion locations, TSDs identified, and the genome sequence that flanks the insertion site, which can be used for primer design (default: 100 bp upstream and downstream of the insertion) for validation of polymorphisms (File S1).

## Discussion

Identifying TE polymorphism among the members of a species is an important component of defining population and genome variation and provides good evidence for activity of a TE family. RelocaTE can be used to quickly identify the locations of TE insertions present in the reference (reference insertions), in the NGS reads only (nonreference insertions), or in both reference and NGS reads (shared insertions) while bypassing the difficulties associated with genome assembly. Importantly, RelocaTE is fast: it can predict insertions of a specific TE in 72× sequence coverage (266,289,512 aligned reads) of a 380-Mb genome in less than 30 min on 64 cores. The speed of processing allows for the analysis of many TEs in many tissue samples, strains, or species in a relatively short period of time. CharacTErizer was developed to determine the genotype of TE insertions sites based on the proportion of flanking sequencing reads that contain the TE and the spanning reads that do not. These software tools provide simple, efficient, and intuitive identification of TE polymorphism within and between individuals using NGS.

The focus of this study, strain A123, is one of four related landraces in which *mPing* is increasing in copy number. As one of the most active TE in a higher eukaryote, it has been of interest to know how *mPing* amplification rapidly diversifies the rice genome. In a prior study of *mPing* movement in landrace EG4 (Naito *et al.* 2009), vectorette PCR was used to specifically amplify *mPing* insertion sites from 24 siblings of a self-cross where the products of each plant were distinguished with barcodes. Sequencing (454, Life Sciences) of the PCR products led to the identification of ∼1600 insertion sites and comparative analysis of individual insertions in each of the 24 plants provided a rough estimate of new (unshared) *vs.* old (shared) insertions.

In contrast, in this study we report a far less laborious and costly protocol in which whole-genome sequencing data from the complete genomes of two siblings were rapidly analyzed and compared with each other and with the reference using RelocaTE. More importantly, analysis of the RelocaTE output by CharacTErizer permitted the accurate identification of many more classes of insertions from old (homozygous) to newer (heterozygous) to the newest events including somatic insertion and excision events. In addition, unlike the prior study that was restricted to PCR products containing sequences flanking *mPing*, here we show that insertion sites of other rice TEs can be easily determined by using RelocaTE to analyze the same collection of NGS reads from A123. An unexpected result was that the strain with the deepest coverage, 72× (A123-2), had fewer somatic events than the strain with 32× coverage (A123-0) and almost the same number as the strain with 19× coverage (A123-1) (Table 1). A possible reason for this result is that the detection of somatic insertions is dependent not only on depth of coverage but also on the amount and type of tissue used for DNA extraction. Almost 60 distinct somatic insertions were detected when an entire leaf of A123-0 was used for DNA extraction. In contrast, a small piece of one leaf each from A123-1 and -2 yielded only 4 somatic insertions in total.

Although RelocaTE may not find every insertion in a genome (particularly those in repetitive regions), the insertions that are identified are well supported due to the stringency of the requirements to call a TE insertion site. These requirements include (i) the unique mapping of reads to a single loci in the reference, (ii) an exact match across the alignment of the reads to the TE, (iii) identification of reads that place both the 5′ and 3′ end of an insertion, and (iv) insuring that only the TE-trimmed end of each read is analyzed for the presence of the TSD. Although paired end data are not required for the algorithm, they can assist in more confident placement of reads, especially in areas with duplicated or low complexity sequence.

Although we developed RelocaTE to determine the insertion sites of the active *mPing* element, it can also be used to discover polymorphic insertion sites of any TE among individuals of a population. RelocaTE is written to be easily configurable to flexibly identify elements with differing TSD sizes and varying stringency in alignment scores to find TE containing reads. The only requirements to identify polymorphic sites are a reference genome, NGS resequencing reads (paired or unpaired), one or more TE sequence, and some information about the TSD (exact sequence or length). This latter parameter is easily obtained for both LTR retrotransposons and for most class 2 families where the TSD length is a conserved feature (Feschotte and Pritham 2007). Furthermore, because the runtime of the tool on a complete dataset is relatively short, multiple TSD lengths can be tested in succession to find a likely candidate. Examples of RelocaTE searches using TEs where only the TSD length is known can be found in the supporting material (Table S4 and File S1). If a more permissive set of insertions is desired, such as in the identification of TEs with similar but not identical TIRs, one supported option is to adjust the number of mismatches allowed when aligning reads to the TE sequence (Table S4).

In summary, when used with NGS reads, RelocaTE and CharacTErizer can detect and characterize TE polymorphism among individuals and accurately identified the rare somatic insertion and excision events from an extremely active TE. Detection of somatic events across the whole genome has not been possible with previous software. RelocaTE and CharacTErizer can be used to identify polymorphic nonreference, reference and shared TE insertion locations to a single base pair resolution for any TE family in any species with a reference genome and NGS from at least one individual. RelocaTE and CharacTErizer can aide in the comparison of TE polymorphisms among individuals enabling the discovery of actively moving TEs and characterizing genetic variation among the individuals in a population. RelocaTE is available on Github (http://srobb1.github.com/RelocaTE/) under the BSD license and short read sequences are deposited under NCBI SRA accession number SRA062826.

## Supplementary Material

Supporting Information
