# Supplementary material for: The Use of RelocaTE and Unassembled Short Reads to Produce High-Resolution Snapshots of Transposable Element Generated Diversity in Rice
Source: G3 (Bethesda). 2013 Jun 1;3(6):949–57. doi: 10.1534/g3.112.005348 (PMC3689806; doi:10.1534/g3.112.005348)
Supplement: Supporting Information [file supp_3_6_949__index.html]

The Use of RelocaTE and Unassembled Short Reads to Produce High-Resolution Snapshots of Transposable Element Generated Diversity in Rice — Supporting Information 

# The Use of RelocaTE and Unassembled Short Reads to Produce High-Resolution Snapshots of Transposable Element Generated Diversity in Rice

## Supporting Information for Robb *et al.*, 2013

**Files in this Data Supplement:**

- Supporting Information - Files S1-S2 and Tables S1-S4 (PDF, 256 KB)
- Table S1 - Somatic excision event classification of non-reference insertions in A123-0 (PDF, 87 KB)
- Table S2 - Excision events with footprints in A123-0 (PDF, 86 KB)
- Table S3 - PCR Primers for non-reference insertion validation (PDF, 92 KB)
- Table S4 - TEs other than *mPing* used for a RelocaTE search in A123-2 (PDF, 98 KB)
- File S1 - RelocaTE output file (.txt, 239 KB)
- File S2 - RelocaTE source code v1.0.1 (.zip, 30 MB)
