# Supplementary material for: The Use of RelocaTE and Unassembled Short Reads to Produce High-Resolution Snapshots of Transposable Element Generated Diversity in Rice
Source: G3 (Bethesda). 2013 Jun 1;3(6):949–57. doi: 10.1534/g3.112.005348 (PMC3689806; doi:10.1534/g3.112.005348)
Supplement: Supporting Information [file supp_g3.112.005348_005348SI.pdf]

G3: Genes | Genomes | Genetics

The use of RelocaTE and unassembled short reads to produce high-resolution snapshots of transposable element generated diversity in rice

Sofia M.C. Robb[*,§,**], Lu Lu[§,**], Elizabeth Valencia[**], James M. Burnette III, Yutaka Okumoto[#], Susan R. Wessler[§,**], and Jason E. Stajich[*,§]
[*] Department of Plant Pathology & Microbiology
[§] Institute for Integrative Genome Biology
[**] Department of Botany & Plant Sciences
University of California-Riverside, Riverside, CA USA 92521
[#] Graduate School of Agriculture, Kyoto University, Kitashirakawa-oiwake Sakyo, Kyoto, Kyoto 606-8502, Japan

Corresponding Author:
Jason E Stajich
Department of Plant Pathology & Microbiology
900 University Ave
University of California, Riverside
Riverside, CA 92521
Office: 951-827-2363
Fax: 951-827-5517

Sequence Accession Numbers: NCBI SRA: SRA062826

DOI: 10.1534/g3.112.005348

File S1
RelocaTE output file

File S2
RelocaTE source code v1.0.1

Available for download at http://www.g3journal.org/lookup/suppl/doi:10.1534/g3.112.005348/-/DC1

Table S1 Somatic excision event classification of non-reference insertions in A123-0

RelocaTE Insertion Position	Avg. Flankers	Perfect Aligned Spanners	Imperfect Aligned Spanners	CharacTErizer Excision Classification
Chr1:1193505..1193507	26.5	1	0	homozygous/excision_no_footprint
Chr1:6432234..6432236	14.5	2	0	homozygous/excision_no_footprint
Chr2:24264669..24264671	14	2	0	homozygous/excision_no_footprint
Chr3:26688131..26688133	12.5	3	0	homozygous/excision_no_footprint
Chr4:9562248..9562250	9	1	0	homozygous/excision_no_footprint
Chr6:22181554..22181556	12.5	3	0	homozygous/excision_no_footprint
Chr6:23960301..23960303	11.5	3	0	homozygous/excision_no_footprint
Chr6:25271453..25271455	7	1	0	homozygous/excision_no_footprint
Chr6:26067399..26067401	12.5	2	0	homozygous/excision_no_footprint
Chr7:3981858..3981860	14.5	4	0	homozygous/excision_no_footprint
Chr7:4528887..4528889	18.5	3	0	homozygous/excision_no_footprint
Chr8:10729277..10729279	17	1	0	homozygous/excision_no_footprint
Chr2:7838215..7838217	17	2	3	homozygous/excision_no_footprint/excision_with_footprint
Chr1:27292708..27292710	23.5	0	2	homozygous/excision_with_footprint
Chr1:28490065..28490067	9	0	2	homozygous/excision_with_footprint
Chr1:32658455..32658457	19.5	0	2	homozygous/excision_with_footprint
Chr1:39904180..39904182	7	0	2	homozygous/excision_with_footprint
Chr2:9438880..9438882	21	0	2	homozygous/excision_with_footprint
Chr3:28577818..28577820	13	0	2	homozygous/excision_with_footprint
Chr4:21249876..21249878	12.5	0	2	homozygous/excision_with_footprint
Chr6:24738718..24738720	10.5	0	8	homozygous/excision_with_footprint
Chr7:19446275..19446277	17	0	2	homozygous/excision_with_footprint
Chr9:12538485..12538487	16	0	3	homozygous/excision_with_footprint
Chr3:8760869..8760871	3	22	5	heterozygous/excision_with_footprint
Chr7:25542894..25542896	2	11	2	somatic/excision_with_footprint

Table S2 Excision events with footprints in A123-0

Insertion Position	Imperfect Aligned Spanners	Aligned Sequences that support	
Chr1:39904180..39904182	2	Chr1:39904177..39904187:	CCA**TAA**GATCA
		INDEL in A123-0:	.......-T..
Chr1:28490065..28490067	2	Chr1:28490062..28490072:	TAG**TTA**TAGCC
			.....A-....
Chr1:27292708..27292710	2	Chr1:27292705..27292715:	TTA**TAA**TGGCG
			.....A-....
Chr1:32658455..32658457	2	Chr1:32658452..32658462:	TTA**TTA**TGGGC
			.....A-....
Chr2:9438880..9438882	2	Chr2:9438877..9438887:	CTA**TTA**GGGAA
			.......G--.
Chr2:7838215..7838217	3	Chr2:7838212..7838222:	GCC**TTA**TGGAC
			.....A-....
Chr3:28577818..28577820	2	Chr3:28577815..28577825:	CCC**TAA**TGGCC
			.....A-....
Chr3:8760869..8760871	5	Chr3:8760866..8760876:	GGA**TAA**GAGCA
			......G-...
Chr4:21249876..21249878	2	Chr4:21249873..21249883:	TCA**TAA**GAAGA
			......G--..
Chr6:24738718..24738720	8	Chr6:24738715..24738722:	CTG---**TAA**GA
			..GGCC.....
Chr7:19446275..19446277	2	Chr7:19446272..19446282:	ATA**TAA**GCGCC
			......G-...
Chr7:25542894..25542896	2	Chr7:25542891..25542901:	ACA**TAA**TGGCC
			.....A-....
Chr9:12538485..12538487	3	Chr9:12538482..12538492:	TAA**TTA**GAGCA
			......G-...

TSDs are in bold.

Table S3   PCR Primers for non-reference insertion validation

Insertion location	primerF	primerR	Annealing Temp	product size (bp)
Chr1:24082898..24082900	TGGTAGCAAATATATTGGGTGTAGTG	CGGTTAAACGTTGAATATGAATAGG	53	191
Chr1:25086305..25086307	GGGCTACAGGCTACAGCATC	CTACGTGTGGTTCGCCTTTC	55	154
Chr1:639973..639975	GTGACATACTACGCACGAAC	AGTCGAAGTTGTGAAGGAAG	53	750
Chr10:17064452..17064454	TGAGCCACGTGTAACAAAGC	TGTATAGGCTTTCCCATTGTCC	55	597
Chr10:22456791..22456793	CAAGCCTTGTGTATCTCTGTGC	AGCATCTTTAAGGGTTCTGGAG	56	451
Chr10:22745428..22745430	TTCTCCCTCCCTTGAGCTTAG	ACTCTCCTTTCTTTCTTCTCCA	55	247
Chr11:17787654..17787656	GCCATGCTCCAAATTAATGG	GCCATGCTCCAAATTAATGG	55	241
Chr11:21964339..21964341	GCCATTCAACTATTAAGGGG	TCTCTCAGTACTATTGCCGTC	52	770
Chr12:19986498..19986500	TCACGACAACTAGCCGATTG	ATTTCTTGCGTGCTTTTTGC	55	155
Chr12: 23741601..23741603	TCTCACTTGTGATTTCAGCC	CATTTTTCATCCTAGGTGGCAG	53	452
Chr12:24042551..24042553	GCCGTCTGATAATTTTTGGC	TCTCCCTTCGTAAAAGTTGAGG	53	505
Chr12:25776835..25776837	CTACTGGTTTGAAACGTAGCCC	GGTGTCAGTGTTACTGTCCAGC	56	452
Chr2:29419743..29419745	GAGAAGCCTGTAAATATGGC	TCGATCTGAACTCCCTACAG	52	818
Chr3:1298989..1298991	AAAACTATATTTCCCCGTGTGG	TGGAAGTAGTGTGACTGTTTAGACG	55	845
Chr3:1429394..1429396	TCTCCCCAAGAGTCAGCTTC	CAACCCAACCCATCATATCC	55	172
Chr3:17269650..17269652	CTGCTTGGGAGTTGATTTCC	CCATTCGACGGTCCATATTC	55	185
Chr3:2737674..2737676	CCATTAGGACGTGGCAAAAG	GCCAGTATTTCACGGCAAAC	55	233
Chr3:30613090..30613092	ATCTGCTGCTGCTGTTTTTG	TTTTTCCCCTATTGTATCACTCTG	55	246
Chr3:33130125..33130127	ATAGCTGCCCTACCCCCTAC	AGAGAGGCCTGAGCTGTGTG	55	190
Chr4:25599162..25599164	CGTTGAACGTGAATAGTGC	ACCATTGAGAAGACTTGGTC	55	768
Chr4:27056954..27056956	TTGTTCTTTGACGCAAGCTG	CTGGCCTCGAAAATGGTTAG	55	151
Chr5:18960105..18960107	GCAGAGATGAACCCTTTTCC	TAGACGTCCCACAGGGTGTC	55	354
Chr5:27073212..27073214	GCAAATCCTACGGAGAAACG	CAAACCCCAAATTGATGTCC	55	151
Chr5:5878603..5878605	CTGGATCCCATAGGCGTTAC	CTGCAATAAGCTGCAAGCAC	55	249
Chr6:11552865..11552867	CACGGCCGCACTTATTTTAG	AACGCCATTTGGTTGATAGC	55	190
Chr6:27559148..27559150	CCATCTCTCTTTTTGACAGC	TTACCATACCACCCCTACAC	55	763

Chr6:29801347..29801349	GGGTTCCCATAGAACTGGTG	GCACTTTCCAGCTAGGTTGC	55	275
Chr7:24773098..24773100	CGCAAAACGTACTTATGACC	CTTCTTGGAGACCATGTAGC	55	1157
Chr7:26909814..26909816	ACCCACCTTACAATGCTCGT	AGGGAAAGGTAGGCGAAATG	55	219
Chr7:29460821..29460823	GGACCAACGGTGCAGTAGAT	GGATGGGCAAATAAGCAGAG	55	206
Chr7:4692621..4692623	CCTCACCCCAATTAGTCATTC	CGCAGCAAGTTAAAAACGAG	56	1174
Chr8:19840876..19840878	TGCGGATAATTACAGGTTGG	CTTATCCGGTTGGTCGAGTG	55	236
Chr8:19850940..19850942	GCGCATAGATGTAAGATGTC	AGGAACTGTACATGGGAGAG	55	751
Chr8:2433355..2433357	AATCATTGGGTTTTGGCAAC	AATCTGTGCGATTTTGAATCC	53	204
Chr8:26602701..26602703	GATTCCCCTCTCCTTTTTGG	AATGGGGAACAGTCCATTTG	55	133
Chr8:28049611..28049613	GTTTAGTTGGTGGAGATTGC	CCCCATACTATGCAAATACC	57	853
Chr8:28359811..28359813	CCCAGCTCTGGATCCTCTC	CCATGGAGTGAGTGCAAATG	55	186
Chr9:11188613..11188615	ACTGGTGATATTCCAACTGC	TCCCTATAAGCAACATAGGC	56	861
Chr9:16182069..16182071	CTGACGCATCATAGAAGTTG	TAATGATGAGGGGGAGAGAGTG	53	502
Chr9:18734559..18734561	CAGGGTTTAAATCGTGATGTCC	GGGAAGGAGGAGGAGGAGTAAG	58	511
Chr9:20462000..20462002	TGTGTAGGATCATCATGTGG	AGTGACGTTTCACCAATAGC	55	781
Chr1: 16327644..16327646	AACCATATCAGGAACGTGGAAC	AGTGGTTTGCCGTAGTTGTCTC	55	455

Table S4 TEs other than *mPing* used for a RelocaTE search in A123-2

TE	TSD Pattern	Insertions Identified								
			0% MM		2% MM		10% MM		20% MM	
		Ref	Shared	Non-ref	Shared	Non-ref	Shared	Non-ref	Shared	Non-ref
Dasheng ORSiTERTOOT0026[a] Retrotransposon	TSD=..... (any 5nt)	302	131	0	156	4	231	26	231	26
RIRE2_LTR[b] LTR/Gypsy	TSD=..... (any 5nt)	233	184	11	190	11	207	42	207	42
nDart1-1 ORSiTETNOOT0013[a] DNA transposon	TSD=........ (any 8nt)	13	10	1	11	1	11	3	11	3
Gaijin[b] DNA/Tourist	TSD=..A (any 2nt followed by an A)	180	111	1	114	1	163	3	163	3
SPMLIKE[b] DNA/En-Spm	TSD=... (any 3nt)	78	75	8	75	14	76	22	76	22
RETRO1_LTR[b] LTR/Gypsy	TSD=..... (any 5nt)	114	78	15	80	17	88	34	88	34
COPIA2-LTR_OS[b] LTR/Copia	TSD=..... (any 5nt)	251	135	0	160	1	233	6	233	6
RIRE3A_LTR[b] LTR/Gypsy	TSD=..... (any 5nt)	88	49	22	51	26	52	35	52	35
TRUNCATOR[b] LTR/Gypsy	TSD=..... (any 5nt)	126	77	0	83	0	84	0	84	0

[a] MSU Plant Repeat Database ID
[b] Repbase ID.
