# Supplementary material for: The Use of RelocaTE and Unassembled Short Reads to Produce High-Resolution Snapshots of Transposable Element Generated Diversity in Rice
Source: G3 (Bethesda). 2013 Jun 1;3(6):949–57. doi: 10.1534/g3.112.005348 (PMC3689806; doi:10.1534/g3.112.005348)
Supplement: Supporting Information [file supp_g3.112.005348_TableS1.pdf]

Table S1 Somatic excision event classification of non-reference insertions in A123-0

RelocaTE Insertion Position	Avg. Flankers	Perfect Aligned Spanners	Imperfect Aligned Spanners	CharacTErizer Excision Classification
Chr1:1193505..1193507	26.5	1	0	homozygous/excision_no_footprint
Chr1:6432234..6432236	14.5	2	0	homozygous/excision_no_footprint
Chr2:24264669..24264671	14	2	0	homozygous/excision_no_footprint
Chr3:26688131..26688133	12.5	3	0	homozygous/excision_no_footprint
Chr4:9562248..9562250	9	1	0	homozygous/excision_no_footprint
Chr6:22181554..22181556	12.5	3	0	homozygous/excision_no_footprint
Chr6:23960301..23960303	11.5	3	0	homozygous/excision_no_footprint
Chr6:25271453..25271455	7	1	0	homozygous/excision_no_footprint
Chr6:26067399..26067401	12.5	2	0	homozygous/excision_no_footprint
Chr7:3981858..3981860	14.5	4	0	homozygous/excision_no_footprint
Chr7:4528887..4528889	18.5	3	0	homozygous/excision_no_footprint
Chr8:10729277..10729279	17	1	0	homozygous/excision_no_footprint
Chr2:7838215..7838217	17	2	3	homozygous/excision_no_footprint/excision_with_footprint
Chr1:27292708..27292710	23.5	0	2	homozygous/excision_with_footprint
Chr1:28490065..28490067	9	0	2	homozygous/excision_with_footprint
Chr1:32658455..32658457	19.5	0	2	homozygous/excision_with_footprint
Chr1:39904180..39904182	7	0	2	homozygous/excision_with_footprint
Chr2:9438880..9438882	21	0	2	homozygous/excision_with_footprint
Chr3:28577818..28577820	13	0	2	homozygous/excision_with_footprint
Chr4:21249876..21249878	12.5	0	2	homozygous/excision_with_footprint
Chr6:24738718..24738720	10.5	0	8	homozygous/excision_with_footprint
Chr7:19446275..19446277	17	0	2	homozygous/excision_with_footprint
Chr9:12538485..12538487	16	0	3	homozygous/excision_with_footprint
Chr3:8760869..8760871	3	22	5	heterozygous/excision_with_footprint
Chr7:25542894..25542896	2	11	2	somatic/excision_with_footprint
