# Supplementary material for: The Use of RelocaTE and Unassembled Short Reads to Produce High-Resolution Snapshots of Transposable Element Generated Diversity in Rice
Source: G3 (Bethesda). 2013 Jun 1;3(6):949–57. doi: 10.1534/g3.112.005348 (PMC3689806; doi:10.1534/g3.112.005348)
Supplement: Supporting Information [file supp_g3.112.005348_TableS2.pdf]

Table S2 Excision events with footprints in A123-0

Insertion Position	Imperfect Aligned Spanners	Aligned Sequences that support	
Chr1:39904180..39904182	2	Chr1:39904177..39904187:<br>INDEL in A123-0:	CCA**TAA**GATCA<br>.......-T..
Chr1:28490065..28490067	2	Chr1:28490062..28490072:	TAG**TTA**TAGCC<br>.....A-....
Chr1:27292708..27292710	2	Chr1:27292705..27292715:	TTA**TAA**TGGCG<br>.....A-....
Chr1:32658455..32658457	2	Chr1:32658452..32658462:	TTA**TTA**TGGGC<br>.....A-....
Chr2:9438880..9438882	2	Chr2:9438877..9438887:	CTA**TTA**GGGAA<br>.......G--.
Chr2:7838215..7838217	3	Chr2:7838212..7838222:	GCC**TTA**TGGAC<br>.....A-....
Chr3:28577818..28577820	2	Chr3:28577815..28577825:	CCC**TAA**TGGCC<br>.....A-....
Chr3:8760869..8760871	5	Chr3:8760866..8760876:	GGA**TAA**GAGCA<br>......G-...
Chr4:21249876..21249878	2	Chr4:21249873..21249883:	TCA**TAA**GAAGA<br>......G--..
Chr6:24738718..24738720	8	Chr6:24738715..24738722:	CTG---**TAA**GA<br>..GGCC.....
Chr7:19446275..19446277	2	Chr7:19446272..19446282:	ATA**TAA**GCGCC<br>......G-...
Chr7:25542894..25542896	2	Chr7:25542891..25542901:	ACA**TAA**TGGCC<br>.....A-....
Chr9:12538485..12538487	3	Chr9:12538482..12538492:	TAA**TTA**GAGCA<br>......G-...

TSDs are in bold.
