# Supplementary material for: The Use of RelocaTE and Unassembled Short Reads to Produce High-Resolution Snapshots of Transposable Element Generated Diversity in Rice
Source: G3 (Bethesda). 2013 Jun 1;3(6):949–57. doi: 10.1534/g3.112.005348 (PMC3689806; doi:10.1534/g3.112.005348)
Supplement: Supporting Information [file supp_g3.112.005348_TableS3.pdf]

Table S3 PCR Primers for non-reference insertion validation

Insertion location	primerF	primerR	Annealing Temp	product size (bp)
Chr1:24082898..24082900	TGGTAGCAAATATATTGGGTGTAGTG	CGGTTAAACGTTGAATATGAATAGG	53	191
Chr1:25086305..25086307	GGGCTACAGGCTACAGCATC	CTACGTGTGGTTCGCCTTTC	55	154
Chr1:639973..639975	GTGACATACTACGCACGAAC	AGTCGAAGTTGTGAAGGAAG	53	750
Chr10:17064452..17064454	TGAGCCACGTGTAACAAAGC	TGTATAGGCTTTCCCATTGTCC	55	597
Chr10:22456791..22456793	CAAGCCTTGTGTATCTCTGTGC	AGCATCTTTAAGGGTTCTGGAG	56	451
Chr10:22745428..22745430	TTCTCCCTCCCTTGAGCTTAG	ACTCTCCTTTCTTTCTTCTCCA	55	247
Chr11:17787654..17787656	GCCATGCTCCAAATTAATGG	GCCATGCTCCAAATTAATGG	55	241
Chr11:21964339..21964341	GCCATTCAACTATTAAGGGG	TCTCTCAGTACTATTGCCGTC	52	770
Chr12:19986498..19986500	TCACGACAACTAGCCGATTG	ATTTCTTGCGTGCTTTTTGC	55	155
Chr12: 23741601..23741603	TCTCACTTGTGATTTCAGCC	CATTTTTCATCCTAGGTGGCAG	53	452
Chr12:24042551..24042553	GCCGTCTGATAATTTTTGGC	TCTCCCTTCGTAAAAGTTGAGG	53	505
Chr12:25776835..25776837	CTACTGGTTTGAAACGTAGCCC	GGTGTCAGTGTTACTGTCCAGC	56	452
Chr2:29419743..29419745	GAGAAGCCTGTAAATATGGC	TCGATCTGAACTCCCTACAG	52	818
Chr3:1298989..1298991	AAAACTATATTTCCCCGTGTGG	TGGAAGTAGTGTGACTGTTTAGACG	55	845
Chr3:1429394..1429396	TCTCCCCAAGAGTCAGCTTC	CAACCCAACCCATCATATCC	55	172
Chr3:17269650..17269652	CTGCTTGGGAGTTGATTTCC	CCATTCGACGGTCCATATTC	55	185
Chr3:2737674..2737676	CCATTAGGACGTGGCAAAAG	GCCAGTATTTCACGGCAAAC	55	233
Chr3:30613090..30613092	ATCTGCTGCTGCTGTTTTTG	TTTTTCCCCTATTGTATCACTCTG	55	246
Chr3:33130125..33130127	ATAGCTGCCCTACCCCCTAC	AGAGAGGCCTGAGCTGTGTG	55	190
Chr4:25599162..25599164	CGTTGAACGTGAATAGTGC	ACCATTGAGAAGACTTGGTC	55	768
Chr4:27056954..27056956	TTGTTCTTTGACGCAAGCTG	CTGGCCTCGAAAATGGTTAG	55	151
Chr5:18960105..18960107	GCAGAGATGAACCCTTTTCC	TAGACGTCCCACAGGGTGTC	55	354
Chr5:27073212..27073214	GCAAATCCTACGGAGAAACG	CAAACCCCAAATTGATGTCC	55	151
Chr5:5878603..5878605	CTGGATCCCATAGGCGTTAC	CTGCAATAAGCTGCAAGCAC	55	249
Chr6:11552865..11552867	CACGGCCGCACTTATTTTAG	AACGCCATTTGGTTGATAGC	55	190
Chr6:27559148..27559150	CCATCTCTCTTTTTGACAGC	TTACCATACCACCCCTACAC	55	763

Chr6:29801347..29801349	GGGTTCCCATAGAACTGGTG	GCACTTTCCAGCTAGGTTGC	55	275
Chr7:24773098..24773100	CGCAAAACGTACTTATGACC	CTTCTTGGAGACCATGTAGC	55	1157
Chr7:26909814..26909816	ACCCACCTTACAATGCTCGT	AGGGAAAGGTAGGCGAAATG	55	219
Chr7:29460821..29460823	GGACCAACGGTGCAGTAGAT	GGATGGGCAAATAAGCAGAG	55	206
Chr7:4692621..4692623	CCTCACCCCAATTAGTCATTC	CGCAGCAAGTTAAAAACGAG	56	1174
Chr8:19840876..19840878	TGCGGATAATTACAGGTTGG	CTTATCCGGTTGGTCGAGTG	55	236
Chr8:19850940..19850942	GCGCATAGATGTAAGATGTC	AGGAACTGTACATGGGAGAG	55	751
Chr8:2433355..2433357	AATCATTGGGTTTTGGCAAC	AATCTGTGCGATTTTGAATCC	53	204
Chr8:26602701..26602703	GATTCCCCTCTCCTTTTTGG	AATGGGGAACAGTCCATTTG	55	133
Chr8:28049611..28049613	GTTTAGTTGGTGGAGATTGC	CCCCATACTATGCAAATACC	57	853
Chr8:28359811..28359813	CCCAGCTCTGGATCCTCTC	CCATGGAGTGAGTGCAAATG	55	186
Chr9:11188613..11188615	ACTGGTGATATTCCAACTGC	TCCCTATAAGCAACATAGGC	56	861
Chr9:16182069..16182071	CTGACGCATCATAGAAGTTG	TAATGATGAGGGGGAGAGAGTG	53	502
Chr9:18734559..18734561	CAGGGTTTAAATCGTGATGTCC	GGGAAGGAGGAGGAGGAGTAAG	58	511
Chr9:20462000..20462002	TGTGTAGGATCATCATGTGG	AGTGACGTTTCACCAATAGC	55	781
Chr1: 16327644..16327646	AACCATATCAGGAACGTGGAAC	AGTGGTTTGCCGTAGTTGTCTC	55	455
