# Supplementary material for: The Use of RelocaTE and Unassembled Short Reads to Produce High-Resolution Snapshots of Transposable Element Generated Diversity in Rice
Source: G3 (Bethesda). 2013 Jun 1;3(6):949–57. doi: 10.1534/g3.112.005348 (PMC3689806; doi:10.1534/g3.112.005348)
Supplement: Supporting Information [file supp_g3.112.005348_TableS4.pdf]

Table S4 TEs other than *mPing* used for a RelocaTE search in A123-2

TE	TSD Pattern	Insertions Identified								
			0% MM		2% MM		10% MM		20% MM	
TE	TSD Pattern	Ref	Shared	Non-ref	Shared	Non-ref	Shared	Non-ref	Shared	Non-ref
Dasheng ORSiTERTOOT0026[a] Retrotransposon	TSD=..... (any 5nt)	302	131	0	156	4	231	26	231	26
RIRE2_LTR[b] LTR/Gypsy	TSD=..... (any 5nt)	233	184	11	190	11	207	42	207	42
nDart1-1 ORSiTETNOOT0013[a] DNA transposon	TSD=........ (any 8nt)	13	10	1	11	1	11	3	11	3
Gaijin[b] DNA/Tourist	TSD=..A (any 2nt followed by an A)	180	111	1	114	1	163	3	163	3
SPMLIKE[b] DNA/En-Spm	TSD=... (any 3nt)	78	75	8	75	14	76	22	76	22
RETRO1_LTR[b] LTR/Gypsy	TSD=..... (any 5nt)	114	78	15	80	17	88	34	88	34
COPIA2-LTR_OS[b] LTR/Copia	TSD=..... (any 5nt)	251	135	0	160	1	233	6	233	6
RIRE3A_LTR[b] LTR/Gypsy	TSD=..... (any 5nt)	88	49	22	51	26	52	35	52	35
TRUNCATOR[b] LTR/Gypsy	TSD=..... (any 5nt)	126	77	0	83	0	84	0	84	0

[a] MSU Plant Repeat Database ID
[b] Repbase ID.
